# Preparation, Scanning and Analysis of Duckweed Using X-Ray Computed Microtomography

**DOI:** 10.3389/fpls.2020.617830

**Published:** 2021-01-08

**Authors:** Dylan H. Jones, Brian S. Atkinson, Alexander Ware, Craig J. Sturrock, Anthony Bishopp, Darren M. Wells

**Affiliations:** ^1^Integrated Phenomics Group, School of Biosciences, University of Nottingham, Nottingham, United Kingdom; ^2^Hounsfield Facility, School of Biosciences, University of Nottingham, Nottingham, United Kingdom; ^3^Division of Plant and Crop Sciences, School of Biosciences, University of Nottingham, Nottingham, United Kingdom

**Keywords:** duckweed, MicroCT (μCT) scanning technology, anatomics, image analysis, petrolatum, anatomical phenotyping

## Abstract

Quantification of anatomical and compositional features underpins both fundamental and applied studies of plant structure and function. Relatively few non-invasive techniques are available for aquatic plants. Traditional methods such as sectioning are low-throughput and provide 2-dimensional information. X-ray Computed Microtomography (μCT) offers a non-destructive method of three dimensional (3D) imaging *in planta*, but has not been widely used for aquatic species, due to the difficulties in sample preparation and handling. We present a novel sample handling protocol for aquatic plant material developed for μCT imaging, using duckweed plants and turions as exemplars, and compare the method against existing approaches. This technique allows for previously unseen 3D volume analysis of gaseous filled spaces, cell material, and sub-cellular features. The described embedding method, utilizing petrolatum gel for sample mounting, was shown to preserve sample quality during scanning, and to display sufficiently different X-ray attenuation to the plant material to be easily differentiated by image analysis pipelines. We present this technique as an improved method for anatomical structural analysis that provides novel cellular and developmental information.

## Introduction

Anatomical phenotyping is a relatively underexplored method of phenotyping, based on high throughput quantification of anatomical features (Gomez et al., 2018; Atkinson et al., 2019). These may include vascular characteristics, organ size and shapes, and tissue arrangement. There are many methods of phenotypic and anatomic data collection (Zhao et al., 2019), that vary in time, cost, resolution, quality of information, and accessibility of technology (Table 1).

**TABLE 1:** Select methods used for anatomical phenotyping.

Method		Live plant possible	Destructive	*In situ*/*Ex situ*	Advantages	Disadvantages
Hand section	Brightfield microscopy (BF)	For a short period	Yes	*Ex situ*	High degree of sample manipulation; High Throughput	Unknown section thickness; varied quality
	Confocal laser scanning microscopy (CLSM)					
Agarose embedding for vibratome Sectioning	BF	For a short period	Yes	*Ex situ*	Wide range of specific section thickness possible; Fast; High Throughput	High attrition rate of embedded samples (for duckweed); Imaging must be done quickly to avoid deterioration
	CLSM					
Wax/Paraffin embedding for microtome sectioning	BF	No	Yes	*Ex situ*	very narrow sections; Fixed samples do not deteriorate	Lengthy fixing process; Loss of live cellular information; Some tissue types cannot be cleared; Lower Throughput
Clearing (ClearSee)	CLSM	No	No	*In situ*	Capable of capturing every cell in an individual in an image stack	Lengthy fixing process; fragile sections; loss of live cellular information; Lower throughput
Scanning electron microscopy (SEM)		No	No	*In situ*	Extremely high resolution 1–20 nm, clear image depth	Lengthy fixing process, loss of live information, samples must be able to handle vacuum pressure.
X-ray computed microtomography (μCT)		Yes	No	*In situ*	Able to discriminate between materials; whole plant; 3D; Multiple 2D projections from a single scan	Lower throughput; Lower resolving power than electron/high resolution light microscopy

**Method**		**Minimum slice**	**ACCESSABILITY**			**Reference**
		**thickness**				
			**Availability**	**Cost**	**Time**	

Hand section	BF	150 μm (Non-standard)	High	Low	Low	St. Croix et al. (2005), Thorn (2016), Huszka and Gijs (2019), Jonkman et al. (2020)
	CLSM	0.05 μm optical slices; 20–120 μm penetration	Medium	Medium		
Agarose embedding”	BF	75 μm	High	Low	Low	Atkinson and Wells (2017)
	CLSM	0.05 μm optical slices; 20–120 μm penetration	Medium	Medium		
Wax/Paraffin embedding”	BF	1 μm	High	Medium	High	Connolly (2005), Hamann et al. (2011)
Clearing (ClearSee)	CLSM	0.05 μm optical slices; 250–500 μm penetration	Medium	Medium	High	Kurihara et al. (2015), Richardson and Lichtman (2015)
Scanning electron microscopy (SEM)		N/A	Low	High	Medium	Zuberer (1984), Fu et al. (2017), Borisjuk et al. (2018)
X-ray computed microtomography (μCT)		0.5 μm	Low	High	Low	Dhondt et al. (2010), Pajor et al. (2013), Tracy et al. (2017), Zhao et al. (2019)

We present a protocol for the anatomical phenotyping of duckweed using X-ray Computed Microtomography (μCT) scanning. Duckweeds comprise a group of aquatic free-floating angiosperms in the family Araceae consisting of five genera: *Spirodela*, *Landoltia*, *Lemna*, *Wolffia*, and *Wolfiella*. Phylogenetic studies have shown that *Wolffia* and *Wolffiella* are the most derived genera, with *Spirodela* being the ancestral (Les et al., 2002). This evolutionary trajectory correlates to a general reduction in both size and morphological complexity, as first observed by Landolt (1986); Supplementary Figures 1A–E. All species comprise of a frond or thallus (Hillman, 1961), a leaf-like structure that in most species floats on the water’s surface, with one face exposed to the air and one in contact with the water (Laird and Barks, 2018).

Members of the most ancestral genus, *Spirodela*, have relatively large fronds (∼10 mm diameter), and possess 7–21 roots per frond (Les and Crawford, 1999). *Landoltia punctata*, formerly considered part of the genus *Spirodela*, is morphologically similar to *Spirodela*, and usually has 1–7 roots per frond, but slightly smaller fronds (Les and Crawford, 1999). For *Lemna*, all species comprise of a single root per frond that varies in length between species (Landolt, 1980). *Lemma* also has significant variation in frond width (∼2–8 mm diameter), length, and thicknesses. The genera *Wolffiella* and *Wolffia* contain the smallest plants, members of both genera are rootless, and have fronds (< 3 mm diameter). One *Wolffia* species, *Wolffia microscopica*, forms a psuedo root (Sree et al., 2015). Duckweed fronds contain parenchymatic tissue containing air spaces between the upper and lower epidermal surfaces, with a large variation in the size and morphology of these air spaces between species (Landolt, 1986). Duckweed primarily reproduce asexually and are extremely fast growing, giving them a wide range of potential applications in industry and agriculture (Hillman and Culley, 1978; Yu et al., 2014; Ziegler et al., 2015). The loss of structures typical of angiosperms in duckweed may elucidate mechanisms of vestigiality and reveal how organ functionality varies between species (Laird and Barks, 2018). A feature of the life cycle in several of the duckweeds is the production of turions. These are frond-like structures, produced vegetatively by a parent frond, that are smaller and lacking roots. They are less buoyant, and sink when they become detached. This is understood to be partly as a result of high starch accumulation (Xu et al., 2018). In this state, duckweed colonies can overwinter or survive other short term stresses, such as surviving under ice in watercourses when frozen, or short term droughts (Kuehdorf et al., 2014). When suitable growth conditions return, turions then rise to the water surface, where they vegetatively produce new individual plants, complete with root and full size fronds (Appenroth, 2002).

To date, the most widely used technique for anatomical quantification of duckweed has been microscopy – either brightfield (light) microscopy or laser-scanning confocal microscopy using autofluorescence and florescent dyes. Such approaches have provided an understanding of the general structures within *Lemna minor* roots (e.g., Melaragno and Walsh, 1976). Techniques such as low temperature X-ray microanalysis (Echlin et al., 1982) and electron microscopy (Kim, 2007) have since been used to investigate the structural anatomy of vascular cells. Confocal images (stained with fuchsin) have been used to visualize the frond surface and identify meristematic pockets (Lemon and Posluszny, 2000). Images of paraffin embedded sections have provided resolution on internal structures allowing identification of cell types. Together these have provided a detailed understanding of the cellular structure of different organs. Although duckweed roots are similar in diameter to those of the widely studied land plant *Arabidopsis*, they are far less permeable to the common fluorescent dyes used in microscopy to image root anatomy, resulting in similar staining procedures showing internal structures far less clearly (Supplementary Figures 1I–K,M). It has been observed that in several aquatic plant roots, there are suberized cell layers in the outer cortex akin to a casparian strip that may act to ‘waterproof’ the root to an extent, this may contribute to the observed poorer penetration of aqueous dyes (Barnabas, 1996). Because of this, it is challenging to observe at the same level of anatomical detail to which we have become accustomed to using other model plants. This problem can be alleviated to some extent by using a detergent to aid the penetration of the dye; however, image quality still falls short of images obtained in other model species. Duckweed roots also regularly contain significant amounts of chlorophyll, further complicating their imaging due to autofluorescence. Similar issues hold true for fronds. In addition to the high chlorophyll content obscuring and interfering with fluorescence, they also possess hydrophobic cuticular surfaces (Borisjuk et al., 2018) which greatly impede the penetration of most fluorescent dyes.

For these reasons, sectioning, fixing and/or clearing provide the most viable options to examine internal anatomy using microscope-based approaches. While these methods produce high quality images of roots and fronds at cellular resolution (Supplementary Figures 1H,L) there are significant drawbacks to both. Sectioning, either of live plants embedded in agarose or of fixed plants embedded in wax or plastic is destructive, and has several limitations in its capacity to capture anatomical data. Firstly, as with all microscopy there are tradeoffs between magnification, resolving power, and focal area of the objective. Secondly, the thickness of the sectioned slice, and the ability of the microscope to penetrate each slice creates an interval between images that can cause critical three dimensional (3D) information loss (Connolly, 2005). Thirdly, live plant sectioning can create a damaged surface region artifact, and finally; *ex situ* visualization of sections can cause a loss of anatomical and cellular context with regards to the plant as a whole.

Using chemical agents to make biological samples more transparent (tissue clearing), works well for certain duckweed species and tissues. Using this in combination with confocal microscopy with tile scanning (iterative imaging using a computer controlled stage able to auto-generate an orthomosaic) and z-step capacity, can create well resolved high resolution images up to the size of a microscope slide, at sub-micron intervals in the Z plane (Fouquet et al., 2015; Kurihara et al., 2015).

Depending on the working distance of the lens, the depth of the sample, and the effectiveness of any clearing and staining, it is possible to obtain complete image stacks of the whole volume of plants for the majority of species. This is an effective method of imaging cellular structures within the majority of duckweed species; however, this does not work well with the more cavernulous fronds (with many gas spaces) due to poor penetration of fixative and clearing agent, or for heavily pigmented regions. It must also be noted that structural damage can occur in samples during the vacuum infiltration steps often required for these protocols. Having to fix and clear samples also creates significant loss of information as well as being time consuming in both the sample preparation and imaging steps, thus limiting the potential for this as a high throughput method of phenotyping.

While a large amount of phenotypic data can be collected with the imaging methods listed in Supplementary Figure 1, several important questions remain unachievable. Confocal microscopy, especially for cleared plants, cannot distinguish non-fluorescent features. Extracellular spaces are prominent anatomical features in duckweed (Supplementary Figures 1F,G, *es*) and phenotypic variation in these spaces is likely to affect the interaction between individuals and the environment, by affecting aspects such as buoyancy. To properly assess the distribution of non-fluorescent features, the methods used must allow for analysis of volume and degree of interconnectedness of gas spaces in the frond and root.

It is for these reasons that the majority of research conducted on duckweed, has been into growth (Lasfar et al., 2007), ionomic parameters (Prasad et al., 2001; Verma and Suthar, 2015; Ziegler et al., 2016; Iqbal et al., 2019), and more recently genomic variation (Wang et al., 2014; An et al., 2019). Little consideration has been given to the effects of anatomy on these traits or vice versa. By better understanding the anatomy of duckweed, this may provide a deeper understanding of duckweed developmental and evolutionary biology, and facilitate the generation of new ideotypes for duckweed crop production. Developmentally and evolutionarily, anatomical analysis presents an opportunity to understand the development of frond structure and reproduction, and to quantify the evolutionary loss of organs and organ complexity. Quantification of environmentally important traits such as the 3D shape of fronds, spatial distribution of roots, density of fronds and turions at different developmental stages would provide basis for a more thorough understanding of how individual plants interact with their aquatic environment through future studies in fluid dynamics. From a production perspective, previous studies have largely focused on growth rates without linking them to anatomical traits (Ziegler et al., 2015) Anatomic analyses may provide novel avenues to improve production efficiency, facilitating selection of duckweed which are more productive in terms of tissue production relative to gas space, therefore yielding more biomass for the area of media occupied. Duckweeds also present a unique opportunity for linking whole-plant anatomy to production given their extremely small body plans.

Over the past few decades advances in μCT, a non-invasive method of 3D imaging, have allowed assessment into agricultural practices, notably in the quantification of structures in the soil matrix and a measurement of plant root architectural responses to environmental conditions (Tracy et al., 2012; Helliwell et al., 2013; Bacq-Labreuil et al., 2018; Rabbi et al., 2018; Schlüter et al., 2018). In more recent years, μCT has also been applied in quantifying a variety of plant anatomical and morphological traits (Table 1, Mairhofer et al., 2017) or to finer detailing of these effects on plant leaf architecture and the structural changes incurred under different stresses in the environment (Mathers et al., 2018; Lundgren et al., 2019). The use of μCT has advanced the understanding of developmental and environmental aspects of plant anatomy including identification of 3D traits that impact mesophyll conductance (Earles et al., 2018) and insights into the evolution of leaf anatomical features in vascular plants (Théroux-Rancourt et al., 2020). In this paper we discuss the advantages of using this non-destructive technique for assessing duckweed genera. Unlike other techniques, μCT allows the scanning of live plants with no prior staining or clearing required. Crucially unlike previous methods described, μCT can distinguish material based on image greyscale level (a function of X-ray attenuation) between air filled and water filled regions. X-ray μCT allows for fast and accurate 3D image analysis, not requiring a deconvolution step as in fluorescence microscopy. The method described here increases the throughput for image acquisition compared with the conventional microscopy-based approaches albeit at slightly lower image resolutions. This study is the first to provide a procedure for assessing the anatomy of aquatic plants using μCT.

## Materials and Methods

All reagents were acquired from Sigma Aldrich (United Kingdom), Petrolatum (Vaseline^®^) from Boots (Boots UK Limited), and consumable equipment purchased from Starlab (United Kingdom) unless otherwise stated. All analysis was performed using Excel 2016 (Microsoft) and Prism 8 (Graph Pad).

### Plant Material and Growth Conditions

The following duckweed accessions were used in this work, provided by the Landolt Collection ETH Zurich.

*Spirodela polyrhiza* 9509

*Spirodela intermedia* 7820

*Landoltia punctata* 7760

*Lemna minor* 7123

*Wolffiella lingulata* 8324

*Wolffia arrhiza* 5684

Duckweed colonies were cultivated in sterile 250 ml conical flasks containing 150 ml of autoclaved N-media (Appenroth et al., 1996) per flask, each inoculated with 5–10 individuals transferred from sterile stock colonies using an inoculating loop, and stoppered with sterile cotton wool. Flasks were then placed in a controlled environment cabinet (Conviron Gen 1000), and grown for 14 days at 21°C, 16-8 photoperiod, and 150 μmol m^–2^ s^–1^ photon flux density.

As duckweed morphogenesis is highly variable; the following criteria for selecting plants for imaging was applied. Individual plants had separated from their mother frond, produced root/s of greater than 3 mm in length (where applicable), had a frond of average or greater size for the population, and had no visible discoloration or deformation of the frond.

### Petrolatum Embedding

**FIGURE 1:**
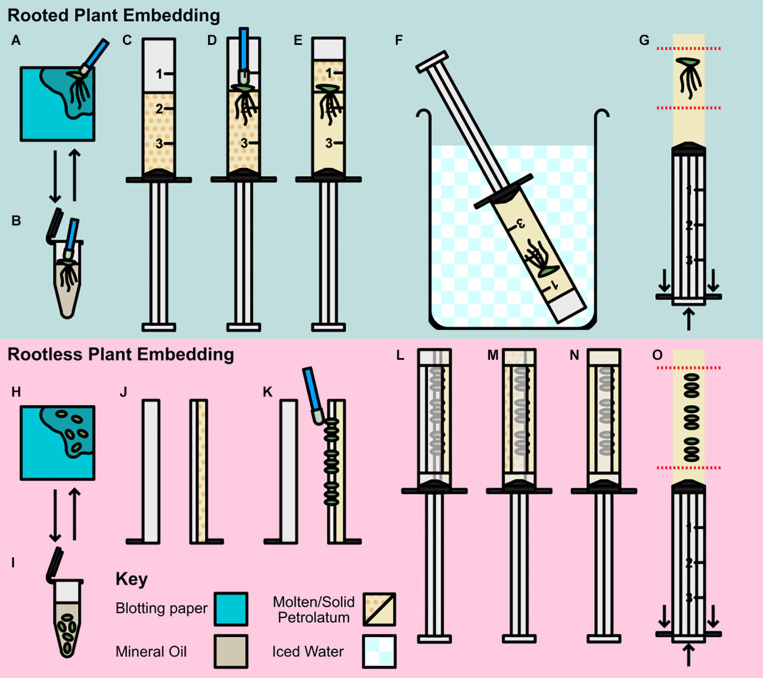
Preparation and embedding of plant material for scanning. **(A–G)** Rooted plant preparation and embedding; **(A,B)** Pre-preparation of plant material; **(A)** Plant transferred by petrolatum coated inoculating loop to blotting paper. **(B)** Plant rinsed in mineral oil then transferred to blotting. **(C–G)** Rooted plant embedding **(C)** Syringe barrel cap cut and removed, and barrel two thirds filled with molten petrolatum. **(D)** Plant transferred to syringe with loop and allowed to partially solidify. **(E)** Additional molten petrolatum added to barrel, covering plant. **(F)** Syringe transferred to ice bath. **(G)** Syringe plunged, and petrolatum core trimmed to region of interest (ROI). **(H–O)** Rootless plant embedding and preparation; **(H,I)** Pre-preparation of plant material; **(H)** Plants removed from media and transferred to blotting paper, **(I)** Plants rinsed in mineral oil and decanted to blotting paper. **(J–O)** Rootless plant embedding **(J)** Syringe barrel cap cut and removed and cut in half along barrel length, two thirds filled with molten petrolatum and allowed to cool. **(K)** Small plants pressed gently into petrolatum with inoculating loop. **(L)** Plunger replaced and barrel resealed with microporous tape. **(M)** Remainder of syringe filled with molten petrolatum. **(N)** Petrolatum allowed to solidify, cooling in ice bath. **(O)** Syringe plunged and petrolatum core trimmed to ROI.

To prepare samples for X-ray μCT scanning plants were embedded in a core of petrolatum inside of modified syringes (Figure 1). Two different approaches were used depending on whether embedding rooted or rootless plants (Without roots, more plants could be embedded in a smaller volume of petrolatum and imaged together, increasing throughput). The following protocol, once components have been prepared, took approximately 3–5 min to prepare a core with a rooted sample embedded, and 5–8 min to prepare a core with between three and nine rootless plants embedded. Unless specified, all steps were carried out at room temperature.

#### Pre-preparation (Figures 1A,B,H,I)

Different embedding methods were used depending on the size of the sample and the presence or absence of roots, however, the pre-preparation method used was the same for all sized plants; plants were removed from media using a petrolatum coated inoculating loop (to avoid physical damage to the frond), and dried of excess media by placing on blotting paper and patted lightly (Figure 1A). Samples were then transferred to mineral oil, and shaken gently, to remove any remaining media and reduce cohesion between roots (where applicable; Figure 1B). Rooted samples were transferred to blotting paper using an inoculating loop (Figure 1A), and rootless samples were decanted with the oil directly onto the blotting paper.

#### Rooted Plant Embedding (Figures 1C–G)

A total of 50 ml of Petrolatum was decanted into a 100 ml glass beaker, and placed in a 55°C incubation oven until molten this was then stored at the same temperature until use. Syringes (3 ml Soft-Ject HSW) were prepared by raising the plunger and cutting the barrel tip off with a razor blade at the shoulder thus leaving just the uncovered open barrel cylinder. Petrolatum was then poured from the beaker into the syringe until two thirds full of molten petrolatum (Figure 1C). Until use, filled syringes were stored at 55°C. Pre-prepared plants, were then transferred to the molten petrolatum and agitated lightly to separate the roots and dislodge any trapped air (Figure 1D). When the petrolatum had solidified enough to hold the frond in place (approximately 30 s), the remaining third of the syringe barrel was filled with molten petrolatum (Figure 1E). The syringe was then placed in an iced water bath until the petrolatum core containing the embedded duckweed was fully solidified (∼3 min; Figure 1F). Once the core was solid, the plunger was depressed, expelling the core which was then trimmed using a razor blade to minimize the regions without sample included for ease of alignment when scanning (Figure 1G). The prepared core was then be stored in the ice bath until ready for use.

#### Rootless Plant Embedding (Figures 1J–O)

Petrolatum was prepared as in the previous section. The tip of the syringe was removed and the barrel bisected along the vertical axis and then taped (microporous tape) along the end of the half barrel. One half of the barrel was then filled to two thirds full of molten petrolatum and allowed to solidify (Figure 1J). Pre-prepared samples were then partially embedded by gently pressing the fronds horizontally with an inoculating loop into the soft petrolatum (Figure 1K). This should only be done enough to secure the frond in place, while ensuring the frond is not pressed hard enough to push the frond through the petrolatum and in contact with the plastic of the syringe. The syringe was then reassembled around the plunger and fastened with micropore tape to seal the seams (Figure 1L). The remaining volume of the syringe barrel was then filled with molten petrolatum by pouring down the unfilled side of the barrel (Figure 1M). Once the petrolatum was partially set (Figure 1N), the syringe was cooled in an iced water bath as in Figure 1F until the petrolatum core had fully solidified. The plunger was then depressed expelling the core; this was then trimmed by hand to the region of interest (Figure 1O). The prepared core was then stored in an ice bath until used. In the case of samples < 3 mm the same process was applied using Falcon Sterological Pipettes (1 ml) instead of the syringes, this allowed for less petrolatum interference during scanning and the ability to achieve higher scanning resolution due to smaller sample width.

### X-Ray Microtomography

**FIGURE 2:**
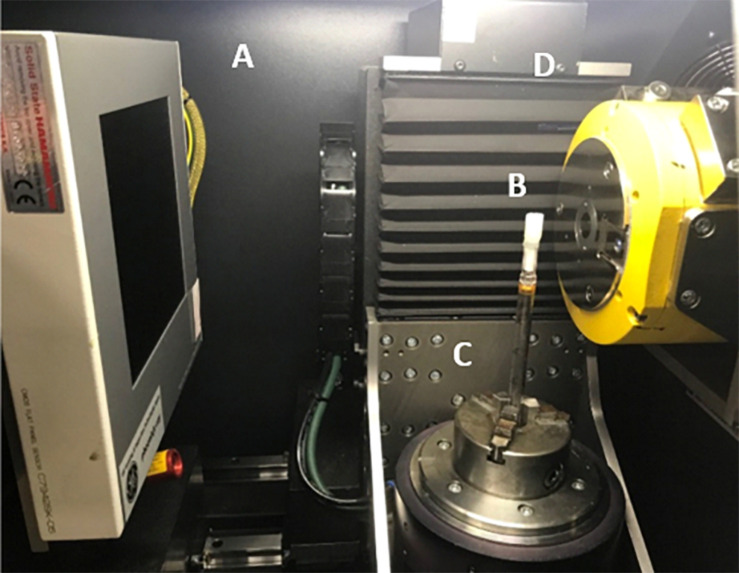
Duckweed embedded in petrolatum core mounted in μCT scanner. **(A)** X-ray detector panel Hamamatsu CMOS Flat Panel (C7942SK-05). **(B)** Petrolatum core, **(C)** Rotation stage, **(D)** X-ray tube (Source).

Each specimen was scanned on a Phoenix Nanotom 180 high resolution X-ray CT system, (Baker Hughes Digital Solutions GmbH, Wunstorf, Germany) at the Hounsfield Facility, University of Nottingham, United Kingdom. Cores extruded from syringes samples (Figure 2B) were mounted on a 10 mm diameter rod using the petrolatum drawn from the edge of the core to hold it in place during the scan on the rotation stage (Figure 2C). Cores prepared in the sterological pipettes were scanned directly by placing the tube within the rotation stage. The scan parameters were optimized to allow a balance between a large field of view and a high resolution (which was dependent on the genera) with the aim to also provide good contrast between petrolatum, plant material, and gas air filled pores. Each sample was imaged using a fast scan procedure with a voltage and current of 70 kV and 100 μA, respectively at a voxel size resolution of between 6–0.8 μm (depending on the genera and sample preparation size), with the specimen stage rotating through 360 degrees at a rotation step increment of 0.25 degrees over a period of 39 min, producing a total of 1,440 projection images were obtained by averaging one frames with an exposure of 1,500 ms each, at every rotation step. The authors would like to note that these settings were optimal in providing the data for high throughput analysis, longer settings can also be used should there be a requirements for better image quality by changing the scan settings to include more projections, increase image timing and increase the number of images captured (*average/skip*) for each projection to reduce movement in the sample during the scan examples of such setting are shown in Table 2. Each scan was then reconstructed using DatosRec software (Baker Hughes Digital Solutions GmbH, Wunstorf, Germany). Radiographs were visually assessed for sample movement before being reconstructed in 16-bit depth volumes with a beam hardening correction of 3. An inline median filter was applied to reduce noise in the image. Reconstructed volumes were then post processed in VG Studio MAX (version 2.2.0; Volume Graphics GmbH, Heidelberg, Germany) see Image Processing.

**TABLE 2:** Standard scan settings used for embedded duckweed cores (of different genera) used at high throughput and an example of high image quality settings.

	X-Ray energy (kV)	X-Ray current (μA)	Projection images	Detector timing (ms)	Image averaging	Image skip	Resolution (μm)	Scan time (min)
Standard settings	70	100	1440	1500	1	0	0.8–6	39
Long scan example	75	120	2440	750	3	2	0.8–6	120

### Sample Stability Testing

Petrolatum embedding was compared to three alternate methods for suitability in maintaining frond stability during μCT scanning. Previously, many small plant μCT methods have used a small sealed chamber (Furuya et al., 2019; Kunishima et al., 2020) in order to reduce desiccation of the samples during scanning. This established method, along with keeping duckweed in N-media, and embedding them in N-media solidified with agarose, was tested.

To determine sample stability, fronds were imaged with a digital camera at 0, 1, and 6 h after preparation (so as to ensure stability exceeded the typical scan time of 1–2 h), with the area of each frond measured at each time point, and observations were made of changes in position and condition of frond over time.

For each treatment, two 24 well trays containing 16 individual plants were prepared according to the different methods and stored under conditions similar to those used in the CT scanner, between imaging intervals.

For plants in air (sealed chambers), plants were removed from media, dried gently on blotting paper, placed individually in wells, imaged, and then covered with laboratory film (Parafilm, Bemis) and stored in darkness until further imaging; Parafilm was removed before and reapplied after imaging.

For liquid media, 2.5 ml of N-media was pipetted into each well, and an individual frond was transferred into each well. Plants were immediately imaged, covered with Parafilm and stored in darkness until further imaging at 1 and 6 h, when Parafilm was removed before and reapplied after imaging.

For embedding in solidified media, agarose (1% w/v) was added to N-media, autoclaved, and allowed to cool to 55°C in an incubating oven. Then 2.5 ml of this molten agarose-media was transferred to 32 wells, and individual plants were then added to each well. Wells were surrounded with ice water, then an additional layer of 0.5 ml of agarose was applied to each well to cover the plants. Trays were imaged, then stored un-covered in darkness until further imaging at 1 and 6 h.

For petrolatum embedding, plants were prepared in a similar method as described above (Figure 1). Plants were pre-prepared in the same way (Figures 1A,B), then transferred to wells filled with 2.5 ml of molten petrolatum, wells were surrounded with ice water, and a minimal amount of petrolatum ∼0.2 ml was then applied to the surface of each frond to cover them completely. Trays were imaged, then stored un-covered in darkness until further imaging at 1 and 6 h.

Frond area at each time point was measured using the magic threshold wand tool in ImageJ (Schindelin et al., 2012), and percentage change in area was calculated in excel. Observations were also made at each time interval of relative position of frond in well, and any changes in condition or appearance of fronds. The frond area at each time point was analyzed in Prism 8 using a two-way analysis of variance (ANOVA) with a Tukeys multiple comparison test, with an *n* = 32 and a *p-*value of 0.05. Percentage area change of fronds over time was calculated in Excel and graphed in Prism 8.

### Alternate Anatomical Imaging

To allow for a comparison of the new μCT method with existing techniques, duckweeds were cleared, sectioned, and imaged using standard techniques described below.

#### Clearing and Whole Root Imaging

Supplementary Figure 1 shows *Lemna minor* roots stained in a similar method to standard *Arabidopsis thaliana* practices (Mellor et al., 2020), immersing the root in fluorescent dye for a short period (3 min), followed by a rinse in purified reverse osmosis (RO) water (1 min) and imaging using a confocal laser scanning microscope with settings specific to the dye used.

Plants were cleared (Supplementary Figure 1H) based on the ClearSee procedure described by Kurihara et al. (2015), modified slightly. As fluorescent markers were not being used, plants were fixed overnight in ethanol and acetic acid (3:1 v/v) rather than paraformaldehyde, as this reduced the toxicity and requirement for vacuum infiltration, which can be damaging to the air spaces. Plants were then rinsed three times in RO water and left for 30 min, then RO water was replaced with ClearSee solution (10% Xylitol, 15% Sodium Deoxycholate, 25% Urea; Kurihara et al., 2015) and left to clear for 2 weeks. Prior to imaging, plants were stained for 1 h with calcofluor in ClearSee (100 μg/ml), and then washed in ClearSee for 1 h. Imaging was carried using a confocal laser scanning microscope (Leica SP5), using a 405 nm diode laser at 12% and hybrid detector with a range of 440–450 nm, gain of 25%, and pinhole of 0.5 AU.

#### Sectioning

The sectioning method presented by Atkinson and Wells (2017) was adapted to better suit the specifics of duckweed anatomy. Polypropylene 15 ml conical centrifuge tubes (Fisher Scientific) were cut perpendicular to the length of the tube into 5 cm lengths of uniform cross section. Micropore tape was used to seal one end of each of the lengths of tube, which were then filled with 5 ml of molten agarose (5% w/v), and allowed to cool to 39°C. Plants were then removed from media with an inoculating loop, dried of excess media on filter paper, and then submerged in the agarose. The number of plants that can be embedded per block is variable and depends on the number of roots per frond. For multi-rooted species (*Spirodela* spp., *Landoltia punctata*), it is the authors’ recommendation to only embed one individual per tube to prevent sample coalescence. For single rooted (*Lemna* spp.), 3–5 plants can be embedded per tube, and for rootless plants (*Wolffiella* spp., *Wolffia* spp., turions), depending on the size of the plants, it is recommended that no more than 30 plants are embedded in a 5 ml block. Embedding multiple plants per block increases the potential throughput of this method, however, can reduce the stability of the block during sectioning.

When embedding plants for root sectioning, for blocks with multiple roots it is desirable for these to be parallel to each other so they can be sectioned in the same plane. To achieve this it was best to hold plants with forceps by the frond, submerge them fully in the agarose to the bottom of the tube, and slowly pull the fronds back toward the surface, which aligned the roots beneath the frond.

To embed fronds for sectioning, to avoid damage to the frond, plants were handled by the root, or Pasteur pipette and inoculating loop for rootless plants. Fronds were submerged in the agarose, and to prevent fronds resurfacing after embedding, they were embedded as the surface of the agarose was setting and monitored until set. To embed many rootless fronds, or turions, after drying can be done by gently stirring them into the cool molten agarose cylinder to distribute them evenly.

Blocks were trimmed by hand at the base prior to mounting to position the sample perpendicular to plane of the desired section. For sectioning a vibrating microtome (7000smz-2, Campden Instruments, Ltd.) was used, the section thickness was 150 μm, blade speed of 1 mm/s and frequency of 65 Hz. Rootless plants may require different settings as the vibration can dislodge the small fronds, thicker slices of ∼250 μm are generally more optimal to avoid sample attrition, however, this will only give a single slice through most rootless duckweed. Additionally, with moderate hand sectioning competency and a sharp blade, similar quality slices could be achieved with a lower attrition rate.

As per Atkinson and Wells (2017) recommendations, for confocal imaging of root and frond sections, sections were stained in fluorescent brightener 28 (calcofluor) solution (0.3 mg/ml) for 1 min then rinsed in RO water for 1 min before imaging on a confocal laser scanning microscope (Leica SP5). This was done using a 405 nm diode laser at 6% and hybrid detector with a range of 440–450 nm, gain of 15%, and pinhole of 0.5 AU.

To image starch content and distribution in fronds and turions, sections were prepared as above, then stained with dilute Lugol’s iodine (25% v/v) for 45 s, rinsed for 1 min in RO water, and imaged on a dissecting brightfield microscope (Zeiss).

### Image Processing

#### 3D μCT Data: VG Studio Max

Reconstructed volumes were post processed in Volume Graphics Studio MAX (version 2.2.0; Volume Graphics GmbH, Heidelberg, Germany). Samples were initially assessed to determine if further filtering was required to reduce image noise. An adaptive gauss filter (smoothing = 1.2, Edge Threshold = 1, Iterations = 1, multiplier = 1) was applied to the data set to reduce the noise and help with image segmentation if this was required. Segmentation was then performed using *surface determination* and *region growing* tools. By selecting greyscale tolerances (determined by sample density and X-ray attenuation) a selection of just plant material could be achieved. This selection could then be saved as a region of interest (ROI), and then following the same procedure, be added to using the region grower tool until the whole plant was selected. The ROI smoothing function of 1 was used to reduce noise artifact before rendering as a 3D model which rotated and clipped to visualize specific regions and structures within the specimen. While largely dependent on processing power of the hardware used, the manual steps of this method took under 5 min to segment a single region of interest.

#### 2D: ImageJ

**FIGURE 3:**
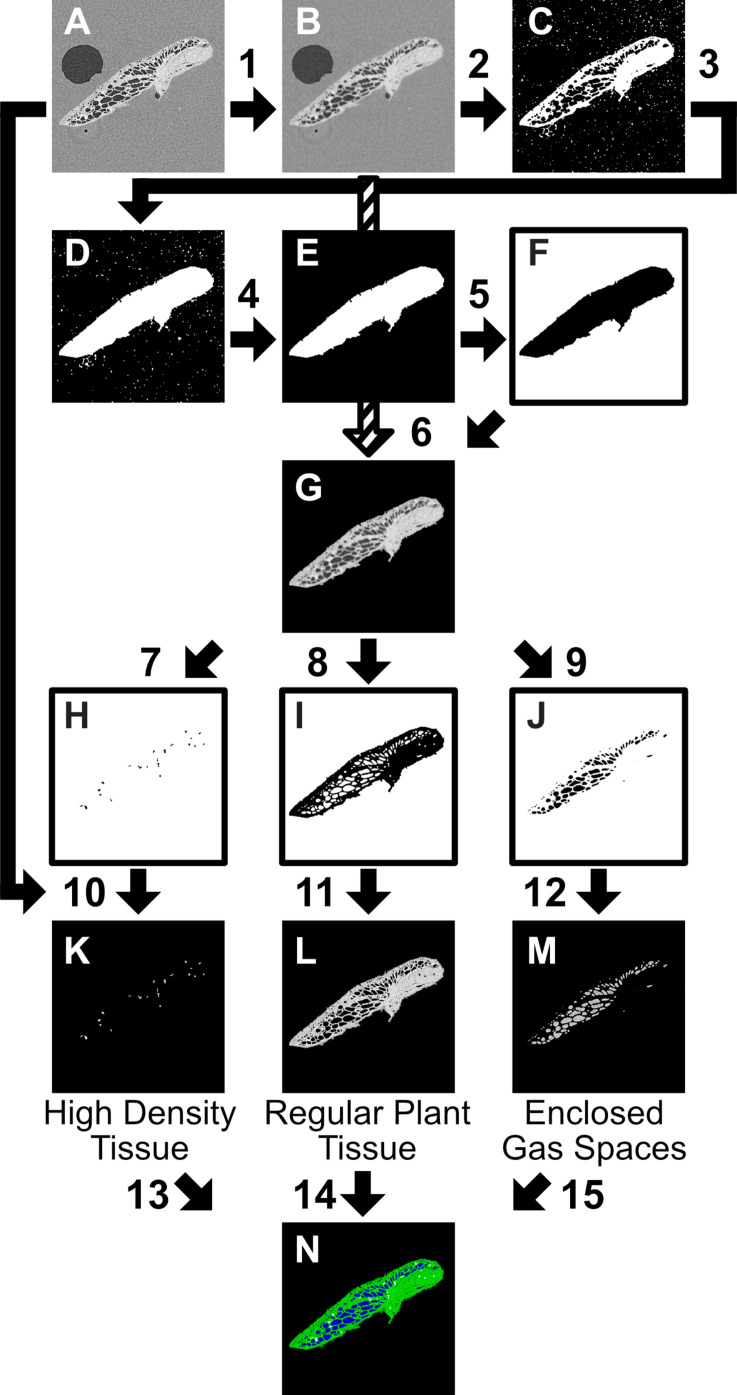
Two-dimensional (2D) slice Image segmentation procedure, carried out in ImageJ, step instructions using ImageJ process commands. The starting point **(A)** is an 8 bit image, or image stack (referred to interchangeably as image). **(1)** Gaussian Blur 2.5 pixel sigma radius **(2)** Threshold 0-155, Invert **(3)** Fill Holes, Open **(4)** Analyze Particles (Pixel size:1000-∞, Circularity 0–1, Show Mask) **(5)** Invert, Dilate, Close, Fill Holes, Erode **(6)** [(**(B)**Add 1)-F] **(7)** Threshold 225–255; Analyze Particles 30-Infinity, Invert **(8)** Threshold 126–224, Invert **(9)** Threshold 1–125, Invert **(10)** A–H **(11)** A–I **(12)** (A: Invert)-J **(13)** Merge Channels: C4 (gray) = ‘**K**’ **(14)** Merge Channels: C2(green) = ‘**L**’; **(15)** Merge Channels:C3(blue) = **‘M’**. All values given are suggested for described scan settings.

Two-dimensional (2D) image stacks were exported in three different projections, XY, ZX, and YX from VGStudioMAX (version 2.2.0; Volume Graphics GmbH, Heidelberg, Germany) from the raw CT data. Image stacks created from VGStudioMAX were exported at resolution thickness 6-0.8 μm depending upon the genera. Different traits within the duckweed were easier to visualize depending on the image plane XY (top down) was best for visualizing gas space interconnectivity and root structure. Whilst ZX (front on) or YX (side on) are best to visualize cross sections along the length of the frond such as Supplementary Figure 1F and that shown in Figure 3.

**FIGURE 4:**
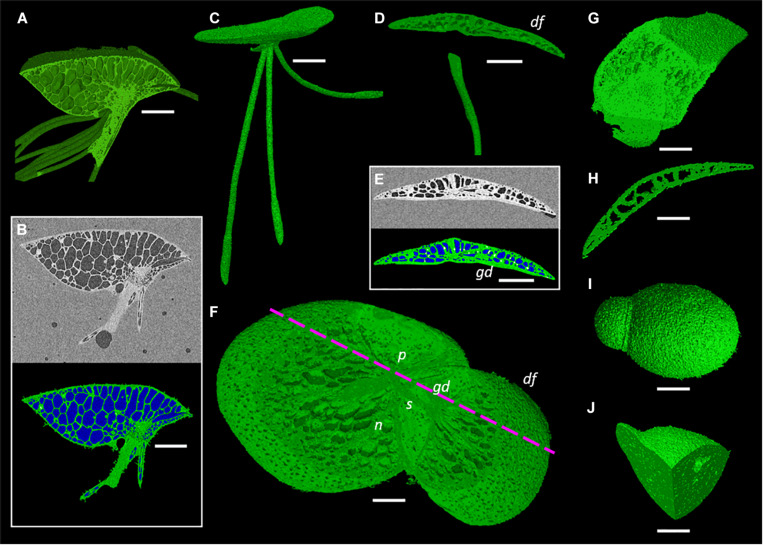
Representative images of duckweed genera showing render capabilities and segmentation of micrographs. **(A,B)** *Spirodela intermedia*, (**A**) Render clipped to midpoint of frond, (**B**) Micrograph and segmentation of micrograph of midpoint slice (White: Starch rich tissue Green: Tissue Blue: Gas), **(C)** Render of *Landoltia punctata*, **(D–F)** *Lemna minor*, (**D**) Render clipped to back half of frond, daughter frond (*df*), granddaughter frond (*gd*), (**E**) Micrograph and segmentation of micrograph, (**F**) Render clipped to partially remove adaxial frond surface revealing stipe (*s*), nerves (*n*), and daughter frond pocket (*p*), mauve dashed line shows clipping plane of **(D**,**E), (G,H)** *Wolffiella lingulata*, (**G**) Render clipped twice, revealing meristem and internal anatomy of daughter frond, (**H**) Render clipped to midpoint showing longitudinal section of frond, **(I,J)** *Wolffia arrhiza*, (**I**) render of whole plant, (**J**) Render clipped twice to reveal internal gas spaces. Scale bars **(A,B,C)**: 1 mm, **(D,E)**: 0.75 mm, **(F):** 0.5 mm, **(G–J)**: 0.3 mm.

**FIGURE 5:**
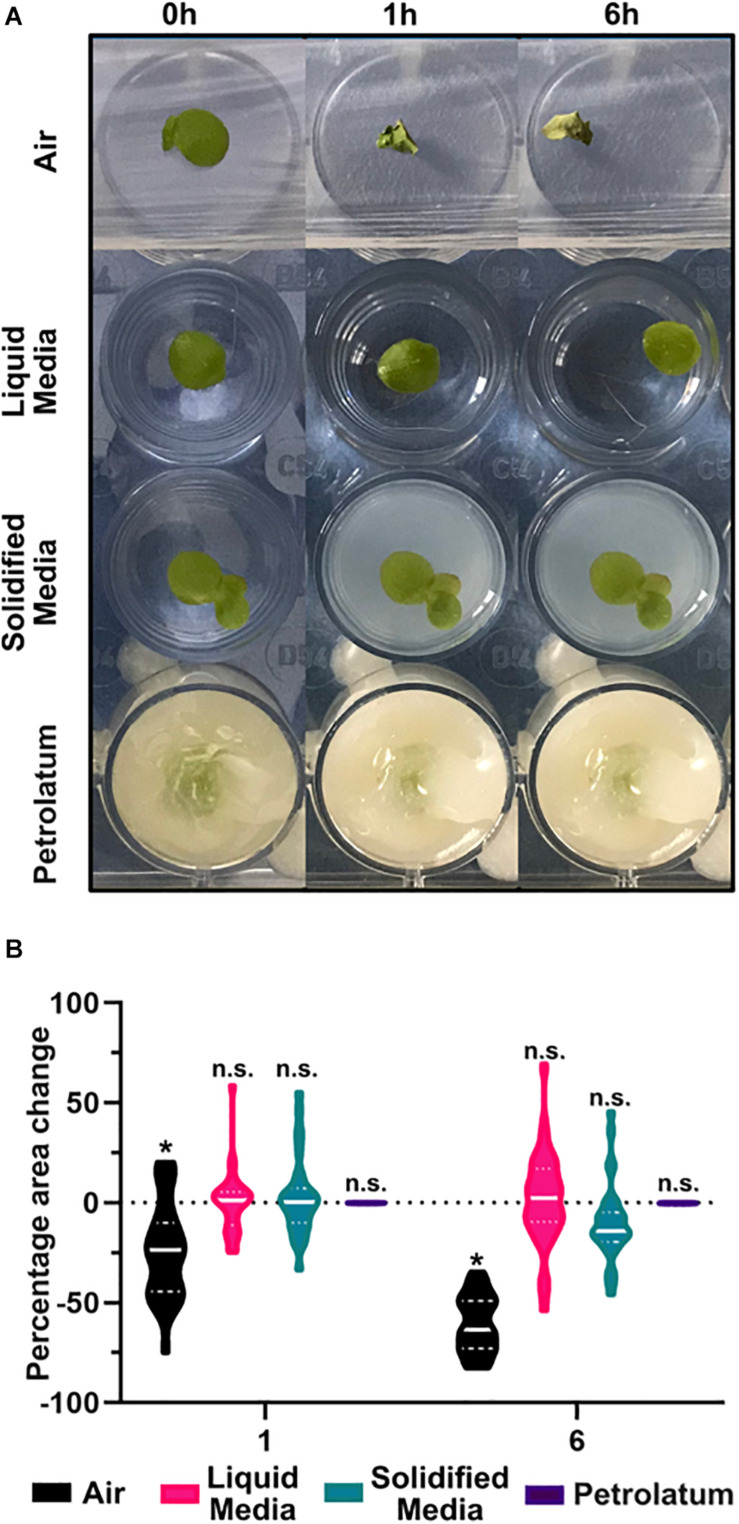
Preparation of samples in petrolatum provides the most stable method for CT imaging. **(A)** Representative images of *Spirodela polyrhiza* condition under different preparation methods over time. **(B)** Percentage change in frond area after 1 and 6 h under different preparation methods, * significant difference in mean area from start point *p* < 0.05, n.s. = No Significance in mean area change from start, *n* = 32 ANOVA with a Tukeys multiple comparison test.

**FIGURE 6:**
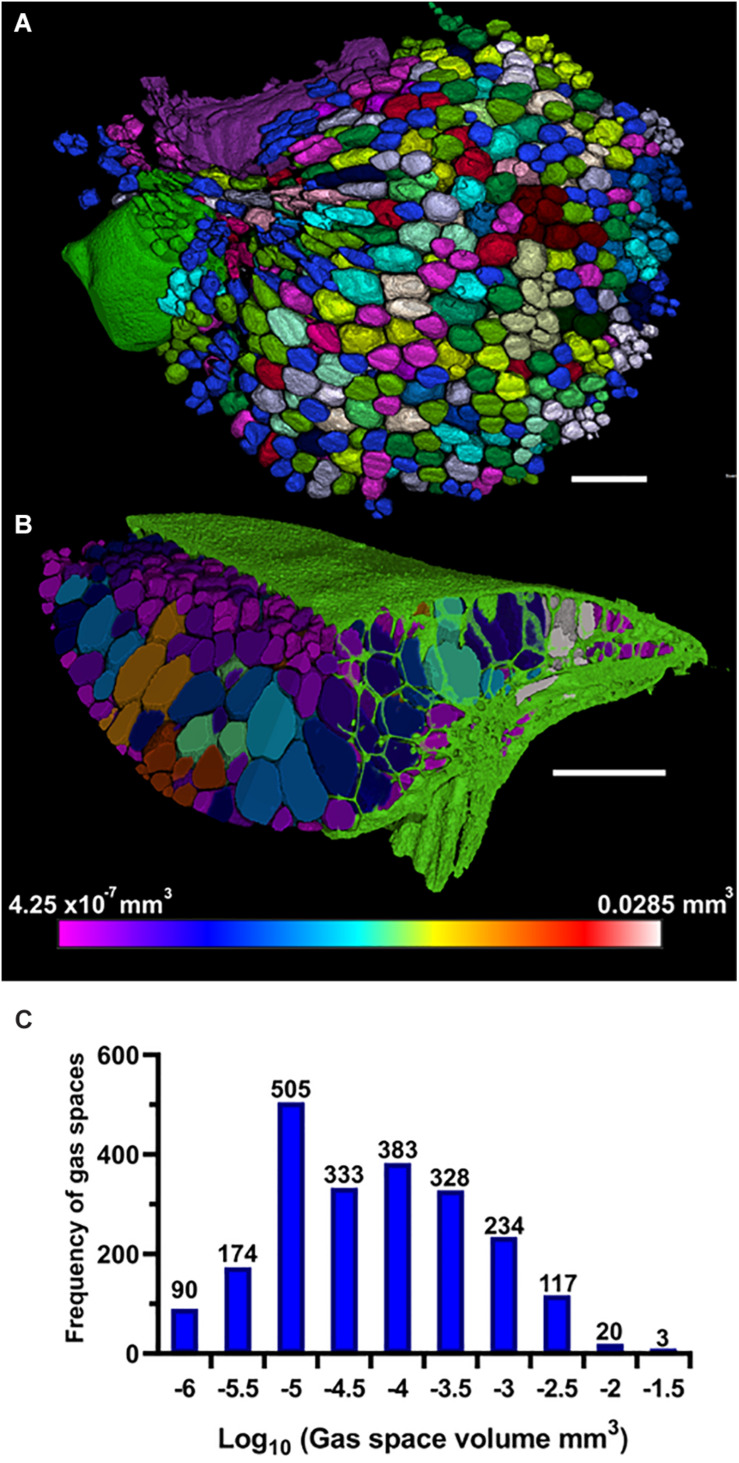
Three-dimensional (3D) particle analysis of *Spirodela intermedia* shows number and volume of each gas space within frond. **(A)** Render showing internal air spaces of *Spirodela intermedia* frond colored by individual gas space. **(B)** Render showing internal air spaces of *Spirodela intermedia* frond, colored by volume, clipped to reveal variation in volume between inner and outer gas spaces within the frond (green). **(C)** Frequency histogram showing distribution in volume of gas spaces within *Spirodela intermedia* frond. Scale bars: 1 mm. Graduated color scale from 4.25 × 10^–7^ mm^3^ to 0.0285 mm^3^.

As within VGStudioMAX, the image stacks were segmented using the greyscale distributions (linked to X-ray attenuation and thus relating to the density of the material) into different categories: plant tissue, gas, petrolatum, and high density regions (thought to be starch deposits based on measurement of particle size (Pankey et al., 1965), following the procedure in Figure 3. The image stacks (referred to interchangeably with image) were loaded into FIJI; then smoothed with a Gaussian Blur (2.5 pixel sigma radius) to reduce noise (Figures 3, 1), thresholded (0–155) and processed using ImageJ binary processing tools (Invert, Fill Holes, Open, Analyze Particles (Pixel size:1000-∞, Circularity 0–1, Show Mask, Invert, Dilate, Close, Fill Holes, Erode) to create an overlay mask containing all plant material and minimal petrolatum or gas outside the plant (Figures 3A–F). This was then inverted and removed from the blurred image, leaving only the features of interest (Figures 3, 2–6).

The features of interest (Figure 3G) were then manually thresholded, using eight-bit image stacks, to different levels to create masks for the desired categories, for example applying a threshold of 1–125 created a mask for enclosed gas spaces, 126–224 for regular plant tissue, and 225–255 for the high density tissue (believed to be the starch deposits). These values can be adjusted to suit the specific image parameters and better align with observed features as required. Full automated thresholds could be applied when processing multiple samples of a similar size. Full set up of this procedure took approximately 10 min, to adjust for variation in threshold values, then subsequent use took under a minute to run for each scan image stack.

For quick visualization of a comparable outcome to the segmentation, a custom lookup table (LUT) using similar values and colorization to those in the pipeline of Figure 3 can be applied (Supplementary Data Sheet 1).

**FIGURE 7:**
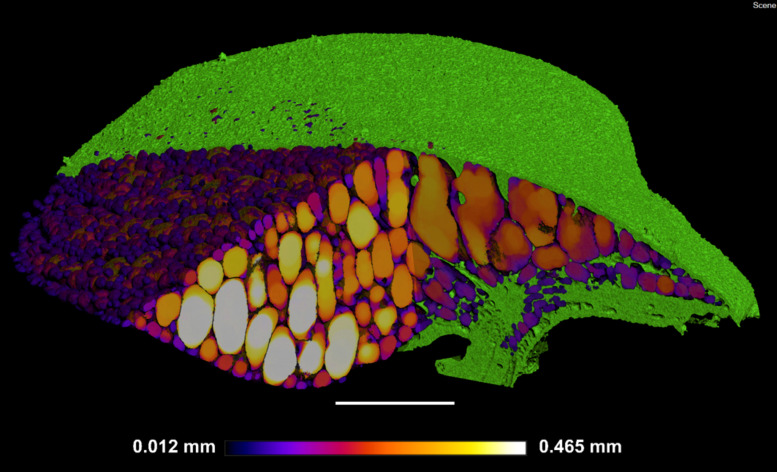
Localized thickness of each gas space within *Spirodela intermedia* frond: Frond tissue in green, gas spaces from Blue–White in graduated scale from 0.012 mm to 0.465 mm in thickness at each point within each gas space in the frond. Scale bar: 1 mm.

A combined approach, utilizing the 2D segmentation procedure for 3D trait analysis was carried out using the ImageJ plugin BoneJ (Doube et al., 2010), as previously used for analysis of gas content of plant material (Lundgren et al., 2019). Particle analysis of the binary masked gas space stack (Figure 3J), outputs measurement of a range of topological features of each distinct gas space (Volume, surface area, Feret diameter, thickness, Euler number), and can generate recolored image stacks, colored according to values of particle traits (number, volume, thickness), which can subsequently be visualized in 3D using ImageJ 3D viewers or with VGStudioMax. Representative 3D renderings of pore number (Figure 6A) and pore size (Figure 6B) as well pore thickness (Figure 7) illustrated by heat map, were constructed in VG StudioMAX (version 2.2.0; Volume Graphics GmbH, Heidelberg, Germany) using Phong rendering tools. Heat map data of the ‘Thickness’ of pores was produced as a function within ImageJ plugin BoneJ which also provided a mean and maximum channel diameter from the image stack. After binarization of the image stacks (Figures 3K–M), use of this plugin was fully automated. Image processing takes from a few minutes to several hours depending on the scan and processing power of the machine used, with the larger the plant and more numerous and interconnected the gas spaces generally the longer the process. Following the 3D particle analysis of internal gas spaces, results were processed in Prism 8. To confirm presence of gas within fronds and roots, pixel value measurements were taken of air inclusions in the petrolatum core, and of gas spaces within frond and root at 50 points within each area using the point tool in FIJI. These measurements were analyzed in Excel with a Student’s *t*-test.

### Turion Composition Analysis

All sunken turions were collected from a colony of *S. polyrhiza* 9509, 2 weeks after inoculation. Of the turions at the bottom of the flask, half were transferred to 50 ml fresh media in a 50 ml falcon tube and stored at 4°C in constant darkness for 8 weeks. The remaining half were transferred to fresh media in a new flask under standard described growth conditions for the same time period. From the new flask, three turions that were floating at the surface of the media, and three that remained sunken at the bottom of the flask were collected. Additionally, three turions from the cold treated set that were not floating were also collected.

All plants were prepared following the rootless plant protocol and scanned using the fast scan μCT settings described. Image stacks were then processed using the described 2D pipeline in ImageJ. Post segmentation, analysis was carried out to determine the percentage composition of each turion based on the three categories of plant tissue, gas, and high density tissue. Total area of each group of was measured for each image stack in FIJI. Based on the known high accumulation of starch in *Spirodela* turions, the size of starch granules in turions, (Xu et al., 2011), the high density of starch, and comparison with iodine stained turions, the high density regions on the scans are likely to be starch deposits. However, other structures have been identified in duckweed fronds that may cause a similarly increased level of X-ray attenuation, such as calcium oxalate or tannin deposits (Mazen et al., 2004; Xu et al., 2018). While iodine staining for a greater duration of time shows that there is starch present throughout the turion, measuring these larger presupposed starch deposits may be a useful proxy for total starch content. To determine turion composition, following measurement of image stacks (Figures 3H–J) in FIJI, total area of each measured component of turions was calculated in Excel, and percentage composition was calculated and graphed in Prism 8, statistical analysis was not performed due to small sample size.

## Results

One species from each duckweed genus was prepared, scanned, and processed using the described methods (Figure 4). Representative scans were rendered into 3D to gauge the efficiency of the preparation and scanning method; showing that across the full range of sizes within the Lemnoideae this method produces high quality images at a relatively fast throughput.

### Method Evaluation

#### Comparison of Embedding Methods

Of the four preparation methods tested, only petrolatum embedding produced consistent usable results. When scanning was performed in either growth media or air, the frond or roots were able to move, and in agarose solidified N-media the plants became deformed, and decreased in area, presumably due to loss of turgor within the core (Figure 5A). These observations were also confirmed in the μCT image data; in addition to a poor contrast between the agarose solidified media and the plant, there appeared to be respiration of the plant, creating a gas pocket around the frond, and significant shrinkage/movement of the frond within resulting in poor or unworkable data.

A significant reduction (*p* < 0.0001) in frond area of 24% was observed after 1 h and significant reduction (*p* < 0.0001) of 60.6% after 6 h when plants were maintained in air (Figure 5B). In liquid media, after 6 h there was a small non-significant increase in frond area with a high degree of frond movement between images. In solidified agar media, there was a small non-significant increase in area after 1 h, and a larger non-significant overall decrease in area after 6 h. When using petrolatum, there was no significant change in frond area at each timepoint.

When exposed to air, fronds dried and shrunk. Despite covering the trays, water still evaporated from the media over the course of the experiment, causing a reduction in well level which caused sample movement and a reduction in the apparent area of frond in the image as the sample moved out of focus. Coupled with the high growth rate of duckweed, this caused variation in appearance of frond size. Similarly to liquid media, the plants in solidified media grew, while the solidified media core shrunk due to evaporation. While this may be mitigated by covering the solidified media core, or embedding within a sealed container, this would increase the diameter of the core and limit the achievable resolution of the scan. Though the mean percentage change in frond area is small for plants on liquid or agarose solidified media, the highly variable results make these methods unsuitable for sample preparation, compared to the highly consistent petrolatum results. In addition to preventing the sample moving, and maintaining area and shape of the sample (Table 3), petrolatum coating maintains the water content of the samples (Korte and Porembski, 2011) and can be stored for several days with no noticeable deterioration of anatomical features.

**TABLE 3:** Results of comparison of embedding methods.

Method	X-Ray contrast (Approx. density difference g/cm^3^)	Maintenance of shape		Movement	
		1 h	6 h	1 h	6 h
Air	Excellent (0.996)	Extremely poor	Extremely poor	High	High
Liquid media	Extremely poor (0)	Good	Poor	High	Very high
Solidified media	Extremely poor (0.001)	Good	Poor	Moderate	Moderate
Petrolatum	Good (0.117)	Good	Good	Low	Low

#### Image Processing Methods

The different image processing methods presented provide a range of options for visualization and analysis depending on the desired information. To visualize the internal structures, segmenting and rendering the plant using the 3D method gives the most flexibility and perspective as one is able to view the plant as a whole (Figures 4C,I) or clip into the render revealing internal structures (Figures 4A,D,F,G,H,J). This can be done aligned to the three projection planes (XY, ZX, ZY), or unaligned, presenting different perspectives to those possible with 2D alone. The plant can also be rotated and clipped to multiple planes to reveal or isolate particular features in the 3D object view (Figures 4G,J). Though very rich in information and capable of enabling measurements of internal structures, this method is the most time consuming in segmentation, costly in processing power, and limited in the rate of measurements possible without additional computational measurement add-on packages to the software.

The 2D freeware analysis pipeline presented provides a higher throughput analysis method that is largely automatable and has a much lower requirement of processing power. It is well suited for analysis of plant composition (tissue/gas), and rapid measurement of segmented features. If properly embedded, specific measurements can be manually taken such as organ length, width, and thickness. Of the scans taken, segmentation of the micrographs agrees with visual assessment of ROIs with a high degree of accuracy. To observe specific traits such as gas space interconnectivity, distribution of nerves, and root anatomy, it is easier to do so by parsing through 2D stacks than clipping through the 3D model as the 2D images are of a single plane (depending on the data set size), and can be done without the need for segmentation.

The method of 3D trait analysis of gas spaces using the 2D segmentation worked well for analysis of the representative scans of *S. intermedia, L. punctata, L. minor*, and *W. lingulata* included in Figure 4, however, the process ran poorly on the *W. arrhiza* and *S. polyrhiza* turions due to the resolution of the scan and the small size of the gas spaces. Using the 3D particle analysis tools in the plugin BoneJ (ImageJ), all internal gas spaces in the frond were measured, providing gas space volume, surface area, thickness, diameter, and Euler number (connectivity), as shown in Table 4. Analyzing this data from the *S. intermedia* scan, the volume distribution of gas spaces can be determined (Figure 6C) and thus statistical analysis can be performed on the uniformity of the frequency both at an intra and inter species level when applied to large experimental programs. The recolored image stack outputs from BoneJ allow for visualization of the number of individual gas spaces in 3D (Figure 6A), and the distribution of differently sized gas spaces throughout this frond. The volume differences between gas spaces shown by the graduated color mapped image (Figure 6B), and the thickness of each gas space shown by the heat mapped image (Figure 7), show that in this individual, larger and thicker gas spaces are located in the middle of the frond, while smaller and thinner gas spaces are found at the surfaces of the frond. This type of quantification will allow for much better understanding and phenotyping of duckweed species when applied to a whole replicated experimental program.

**TABLE 4:** Results of 3D particle analysis of gas spaces within representative fronds scanned.

Line	Number of	Vol. (mm^3^)				Surface area (SA) (mm^2^)			Euler (χ)		Thickness (mm)	
	gas spaces											
		Min.	Median	Max.	Total	Min.	Median	Max.	Mean	Std. err.	Mean	Std. err.
*S. intermedia*	2187	4.25E–07	0.00003	0.02853	0.5822	0.00447	0.02763	4.963	0.9186	0.02292	0.08454	0.001251
*L. punctata*	3167	1.97E–07	1.1E–06	0.001	0.01173	0.000267	0.004	1.261	0.982	0.002526	0.02346	0.000272
*L. minor*	926	2.76E–07	4.53E–06	0.00105	0.01792	0.00159	0.00795	0.7155	0.9633	0.007848	0.0313	0.000209
*W. lingulata*	228	1.13E–08	5.65E–07	0.00042	0.00347	0.00019	0.00284	0.5657	0.9167	0.03213	0.03082	0.000523

Following conversion of the image stack to 8-bit, the custom LUT (Supplementary Data Sheet 1) provides an option for rapid visualization of an approximate segmentation of the 2D image stacks but does not enable accurate automated measurement. This requires minimal processing power, and still allows manual measurements to be taken.

### Compositional Determination

**FIGURE 8:**
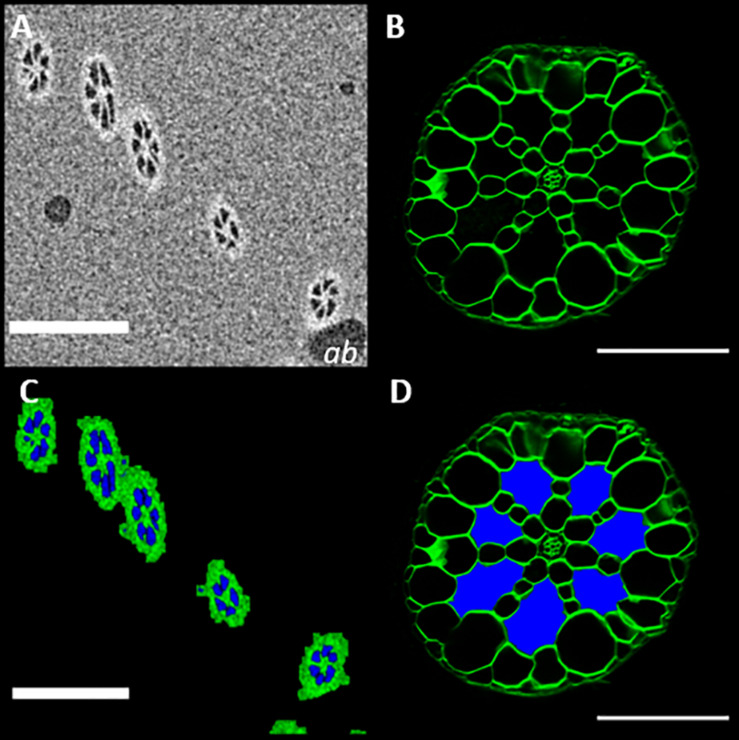
MicroCT imaging confirms that the extracellular spaces seen in root sections are filled with gas **(A)**μCT micrograph of *Spirodela intermedia* root embedded in petrolatum, with air bubble inclusions (**ab). (B)** Confocal image of a *Spirodela intermedia* root section, 150 μm thick, stained with calcofluor **(C)** Segmentation of **(A)** using the method described in Figure 3, **(D)** Recolored root section **(B)** showing the classification of extracellular spaces based on segmentation in **(B),** Scale Bars: **(A,C)** = 200 μm **(C,D)** = 50 μm.

In addition to enabling the acquisition of three-dimensional anatomical data, μCT scanning can also support other methods of anatomical phenotyping. Sections of *S. intermedia* roots (Figure 8B), showed large extracellular voids. Though these were identifiable as aerenchyma based on understanding of root anatomy (Jung et al., 2008), confirming that these are in fact interconnected gas filled channels, was not possible with sectioning alone. Measurement of the image pixel values for the air bubble inclusions within the petrolatum, and the values observed inside the root channels and frond are similar enough that we can say with confidence (*p* = 0.0154) that the extra-cellular spaces in the root are gaseous in the samples we observed. This agrees with the segmentation method described in Figure 3, as shown in Figure 8C. Using this information, the extracellular spaces in Figure 8B, can be labeled and recolored as air spaces (Figure 8D).

**FIGURE 9:**
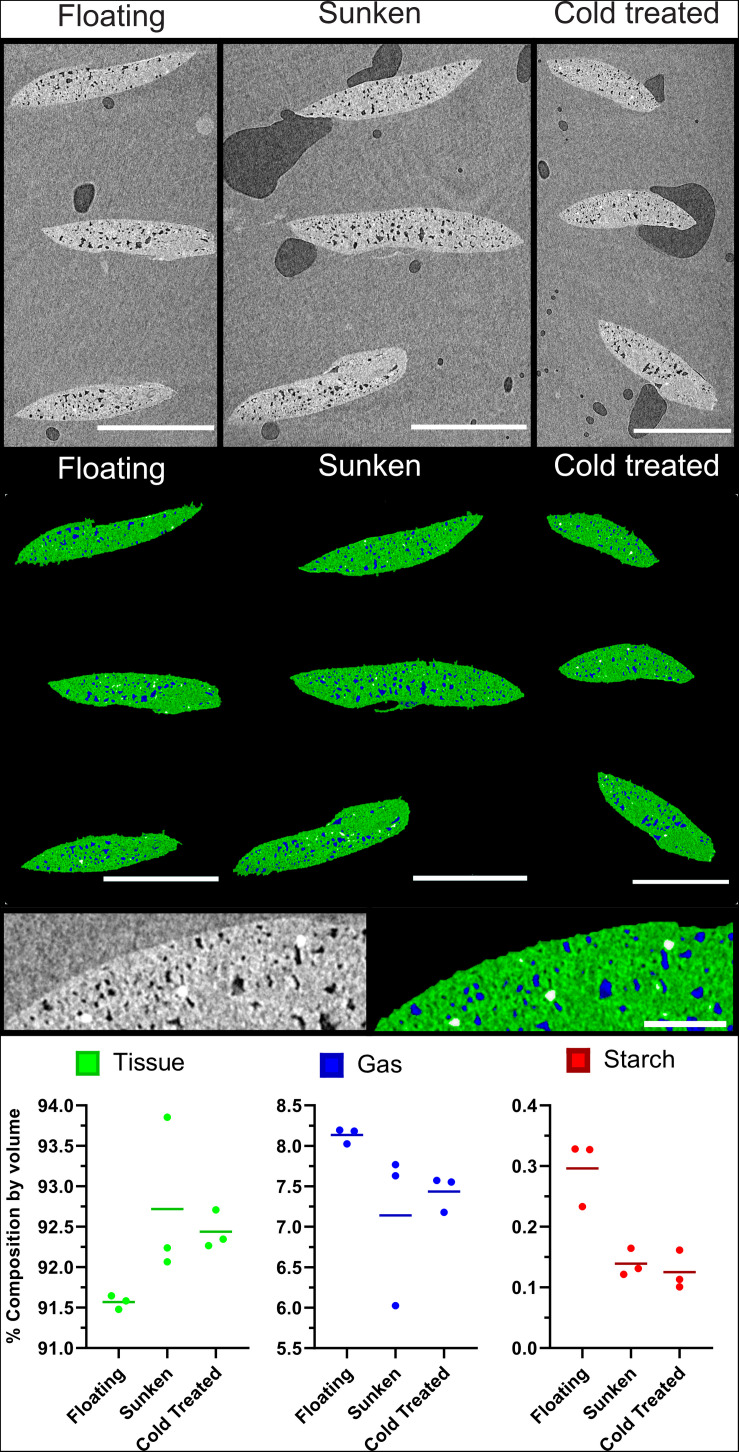
Buoyant *Spirodela polyrhiza* turions contain a higher percentage of gaseous extracellular space than those that are submerged. **(A–C)** Mid slice of three μCT micrographs of stacks of three turions, collected under differing conditions. **(D–F)** Segmentation of images from **(A)** following steps in Figure 3. Green: Tissue, Blue: Gas, White: Starch **(A,D)** Turions floating at surface, **(B,E)** Turions sunk to bottom of flask, **(C,F)** cold treated turions showing petrolatum (*p*), Starch (*s*), Tissue (*t*), and gas spaces (*gs*). **(G)** Expanded micrograph of Cold treated turion. **(H)** Expanded segmentation of micrograph of Cold treated turion showing petrolatum (*p*), Starch (*s*), Tissue (*t*), and gas spaces (*gs*) recolored as determined by segmentation. **(I)** Composition values of each turion set under each treatment as determined by processing and analysis pipeline. Dots represent data points, horizontal line the mean value. Scale Bars **(A–F)**: 1 mm, **(G,H)**: 0.1 mm.

Further to the confirmation of gas spaces, μCT can be used as a method to evaluate differences in material composition. The imaging and analysis pipeline was shown to be effective in evaluating differences between collection variants of duckweed turions. Figures 9A–C show three representative images from the middle slice of XZ image stack of sunken, floating, and cold-treated turions; whilst Figures 9D–F shows the segmentation of these slices using the 2D segmentation method described in Figure 3. Quantification of turion composition shows a differing range of percentage composition of tissue, gas, and starch between collection variants. Although significance testing was not possible due to the small sample size, the scans showed a lower proportion of tissue and a higher proportion of gas in the floating turions than the sunken or cold treated turions (Figure 9I).

## Discussion

Of the methods investigated for anatomical phenotyping of duckweed, the only technique that allowed live whole plant imaging with the capability to visualize internal anatomical features was μCT scanning. To enable μCT imaging of duckweed, samples must be prepared in a way that meets the criteria outlined within this manuscript (maintain turgor, limit unwanted movement, and have sufficient contrast). The method of plant preparation tested that met all of these needs was embedding in petrolatum. Other workable techniques may exist; but the method presented here is quick, inexpensive, and easy to use, and produces high quality results.

To optimize the image acquisition during μCT scanning, it was best to position the sample as close to the x-ray tube as possible, as this increased the resolution of the scan allowing for better observation of anatomical features. The size syringe used to prepare the petrolatum core can be changed depending on the size of the sample. For the majority of duckweed lines, a 3 ml syringe (10 mm diameter) is well suited, smaller sizes may be used, but the viscosity of the molten petrolatum may make liquid handling more difficult. For plants that are too large or small, there are alternatives to embedding the whole plant in a cylindrical core of petrolatum. For small rootless plants, similar to Figure 1K, plants can be pressed gently into a narrower half pipe filled with petrolatum, rather than filling in and extruding a core, and then wholly dipped or coated in petrolatum. This gives narrower diameter profile, allowing a sample to be closer to the X-ray source and increasing geometric magnification of the scan. For larger aquatic plants, such as *Pistia stratiotes*, roots may be embedded in a larger syringe (10 ml) as in Figure 1D, then the leaves can be dipped in molten petrolatum to coat the surfaces. Cores prepared following the steps in Figure 1 are unlikely to have any parts of the plant exposed after the core is extruded, however, if they do, dipping in molten petrolatum will seal any remaining uncovered regions. If not intending to examine root anatomy, roots may be removed from rooted genera, and plants treated as rootless for the described protocols, increasing throughput.

The advantage of taking care to remove media from, and reduce cohesion between the roots when embedding plants in molten petrolatum (as opposed to simply sandwiching plants in, or coating plants with petrolatum) is that it allows the roots to spread and position themselves relative to the frond as they would in media. This makes it easier to segment and measure individual roots, as well as to accurately visualize the morphology of the plant as a whole, in a way more similar to how it would appear ‘*in aqua.’*

Other phenotyping methods may be better suited to the measurement of specific traits, given the limited availability and potential high cost associated with μCT. Much of the phenotypic data gathered from a μCT scan, can also be collected with other methods, albeit in a much slower and time consuming manner. 3D geometry of the plant can be obtained without the use of tomography scanning, especially easily due to the simplified body plan of the majority of species, and internal anatomical data can be collected through sectioning and microscopy. Clearing and mounting whole plants can give gross anatomical and cellular data in tandem, with either confocal microscopy or lightsheet scanning. Where higher resolution may be required, various techniques utilizing electron microscopy may be used (Melaragno and Walsh, 1976). Of the methods investigated, identification and quantification of all the gaseous spaces and tissue within the plant is only currently possible with μCT scanning, and the richness of the data coupled with the high throughput imaging and analysis methods presented make this the best method for structural anatomical phenotyping of duckweed of the methods investigated. As shown in Figure 8, to gain anatomical insight on a structural and cellular level is through a combination of μCT scanning and confocal microscopy.

Of the image processing and analysis methods, a combined approach of 2D and 3D visualization, segmentation and analysis tools was most effective. The most dynamic and illustrative visualization possible was in using the designated 3D software, though manual measurements were easiest to obtain from 2D image stacks (although there are software upgrade packages within VGStudioMAX which can provide 3D image measurements). For segmentation, there were a variety of tools in the 3D software package that enabled the most accurate segmentation of anatomical features, however, this can be time consuming and required a high degree of skill. The 2D method was also highly accurate but took less time, is amenable to automation, and can be optimized to specific scans and sample sizes with simple adjustments. For post-segmentation analysis, all measurements presented of 2D and 3D features used ImageJ, though it would have been possible to do this using add-on packages for the 3D software. At any point in the 2D processing, the image stacks could be loaded back into the 3D software for visualization or to use the different tools available. While not presented here due to limitations of the resolution of scans available, longer scans at a higher resolution may allow measurement of pore size and interconnectivity of the smaller gas spaces present in *W. arriza* and *S. polyrhiza* turions.

Results from turion composition analysis suggest that the mechanism by which position in the water column may be moderated is through variation in anatomical structure and composition. The analysis of air spaces was comparable to previous analyses that have estimated the cellular volume taken up by aerenchyma air spaces in turions in *S. polyrhiza* at between 9–15% using a combination of sectioning and TEM (Kim, 2013). The presented methodology confers several advantages. Firstly, μCT allows the full 3D turion to be investigated and so composition does not need to be inferred from a few slices. Secondly, as this technique is non-destructive, it could be applied to the same turion at multiple stages during dormancy to observe changes over time. Further work is needed to test whether the increased volume of gas filled space within the turion is causal for its rise from the bottom of the water body. Here, we show that using the provided methods it is possible to find variation in anatomical structures. Identification and quantification of different tissue composition is feasible based on visual comparison of the size and distribution of the high contrast regions seen within turions in the CT scans, and sections of iodine-stained turions. The high concentration of these regions in turions compared to other lines and tissues suggests these may be starch deposits (though there may be other materials present in different tissues and species that cause a similar level of X-ray attenuation and further work is required to confirm composition). Previous analysis of turions through sectioning have reported the presence of starch granules (Appenroth et al., 2011). Through isolation of these starch grains, SEM has been used to show that these start to reduce in size under exposure to high light, as starch is broken down into lower molecular weight carbohydrates (Appenroth et al., 2011). It will be interesting to see how this breakdown of starch is associated with the small differences in air spaces that we observed in the buoyant versus submerged turions. Out of all the methods discussed, μCT scanning provides the best way of obtaining 3D structural information, as duckweed turions are heavily pigmented and do not clear well, whilst their small size makes them difficult to section using high throughput approaches.

A further application of this method could be to investigate the importance of frond composition from an industrial perspective. Globally, duckweed is becoming a more common food and feed product. For producers, understanding relative anatomical composition of fronds may enable selection of more productive duckweed varieties. 3D analysis of plant morphology would also aid in our understanding of how different duckweeds are adapted to their relative ecological niches and how anatomy affects interaction with their highly dynamic aquatic environment.

In summary, we present a protocol for the preparation and μCT scanning of duckweed (and other potential aquatic species) at reasonably high throughput that produces high-resolution 3D anatomical and compositional information of live specimens.

## Data Availability Statement

The original contributions generated in the study are included in the article/Supplementary Materials, further inquiries can be directed to the corresponding author.

## Author Contributions

DJ and BA contributed equally to the completion of this manuscript. DJ, BA, and DW wrote the manuscript. BA developed CT scanning protocols, performed all CT scans and 3D segmentation, and provided guidance on pre and post scanning procedures. DJ developed methods and carried out experiments. AW cultivated plant material and carried out confocal imaging. CS provided guidance and technical expertise on scanning and analysis. AB acquired plant material, secured funding, founded University Of Nottingham duckweed research group. DW secured funding, provided guidance and help throughout writing process, as well as teaching and supervision. All authors contributed to the article and approved the submitted version.

## Conflict of Interest

The authors declare that the research was conducted in the absence of any commercial or financial relationships that could be construed as a potential conflict of interest.

## Supplementary Material

Supplementary Figure 1 Comparison of duckweed anatomy using conventional microscopy approaches. **(A–E)** Representative brightfield images of each duckweed genera **(A)** *Spirodela intermedia*, **(B)** *Landoltia punctata*, **(C)** *Lemna minor*, **(D)** *Wolffiella lingulata*, **(E)** *Wolffia arrhiza* **(F,G)** Cross section of *S. intermedia* **(F)** Frond, **(G)** Root based on sectioning with a vibratome followed by staining with calcofluor and confocal microscopy. **(H)** Maximum projection of cleared *L. Minor* stained with calcofluor. **(I–M)** Comparison between different staining methods for analyzing Root tips. **(I)** Maximum projection image of *L. minor* root stained with Calcofluor (CF). **(J)** Maximum projection of *L. minor* stained with Propidium Iodide (PI)**, (K)** Maximum projection of *L. minor* stained with PI & Tween 20. **(L)** Maximum projection of *L. minor* cleared and stained with CF. **(M)** *Arabidopsis thaliana* (Col-0) stained with PI for comparison. Scale Bars: **(A–C)** 5 mm, **(D,E)** 1 mm, **(F,H)** 500 μm, **(G)** 50 μm, **(I–M)** 100 μm **es** = extra-cellular Space.

Supplementary Data Sheet 1 Lookup Table.
