# Supplementary figures and images for: Preparation, Scanning and Analysis of Duckweed Using X-Ray Computed Microtomography
Source: Front Plant Sci. 2021 Jan 8;11:617830. doi: 10.3389/fpls.2020.617830 (PMC7820725; doi:10.3389/fpls.2020.617830)

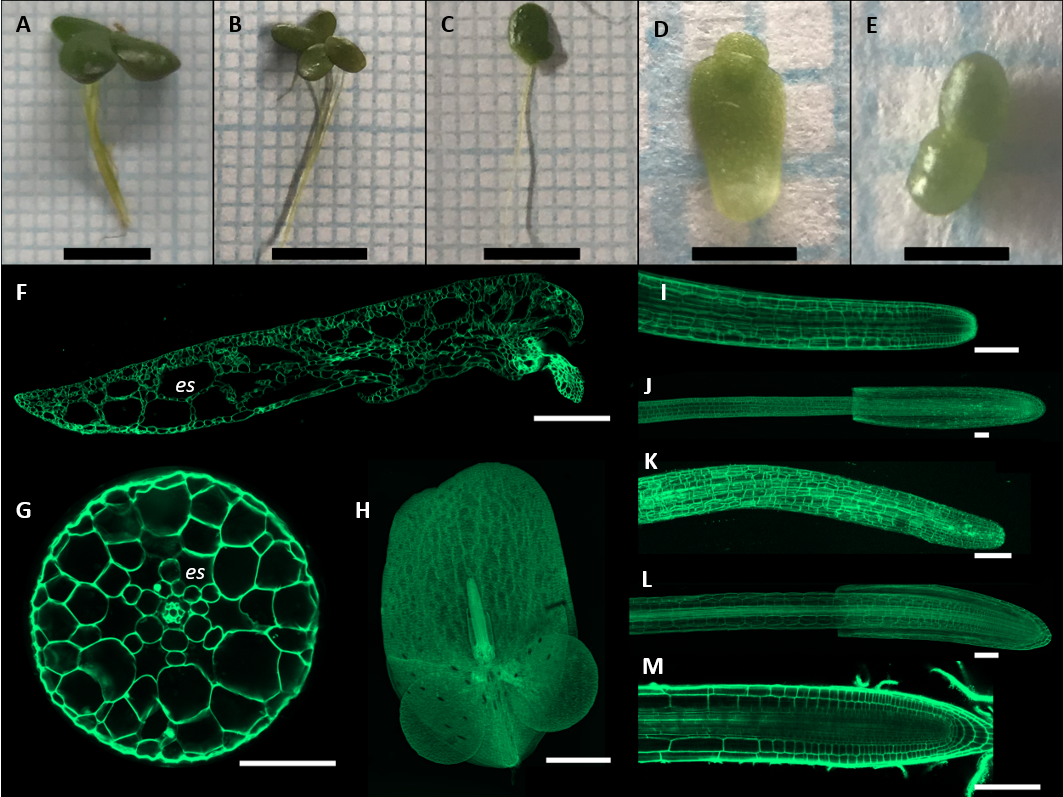

Supplement: Supplementary Figure 1 — Comparison of duckweed anatomy using conventional microscopy approaches. (A–E) Representative brightfield images of each duckweed genera (A) Spirodela intermedia, (B) Landoltia punctata, (C) Lemna minor, (D) Wolffiella lingulata, (E) Wolffia arrhiza (F,G) Cross section of S. intermedia (F) Frond, (G) Root based on sectioning with a vibratome followed by staining with calcofluor and confocal microscopy. (H) Maximum projection of cleared L. Minor stained with calcofluor. (I–M) Comparison between different staining methods for analyzing Root tips. (I) Maximum projection image of L. minor root stained with Calcofluor (CF). (J) Maximum projection of L. minor stained with Propidium Iodide (PI), (K) Maximum projection of L. minor stained with PI & Tween 20. (L) Maximum projection of L. minor cleared and stained with CF. (M) Arabidopsis thaliana (Col-0) stained with PI for comparison. Scale Bars: (A–C) 5 mm, (D,E) 1 mm, (F,H) 500 μm, (G) 50 μm, (I–M) 100 μm es = extra-cellular Space. [file Image_1.tif]
